# SUCCESSES AND CHALLENGES OF PROGRAMS FOR PEOPLE WHO LIVE ALONE WITH DEMENTIA

**DOI:** 10.1093/geroni/igae098.1686

**Published:** 2024-12-31

**Authors:** Yoon Chung Kim, Michael Lepore, Kate Gordon, Lirisha Tuladhar, Heather Menne

**Affiliations:** University of Maryland Baltimore, Baltimore, Maryland, United States; University of Massachusetts Amherst, Amherst, Massachusetts, United States; Splaine Consulting, Columbia, Maryland, United States; Miami University, Oxford, Ohio, United States; Miami University, Oxford, Ohio, United States

## Abstract

Approximately a quarter of older adults with dementia or cognitive impairment live alone in the US. To address the needs of people living alone with dementia (PLAWD), the Administration for Community Living requires PLAWD-focused services in its Alzheimer’s Disease Program Initiative (ADPI). We examined final reports from ADPI grantees that served PLAWD to determine the types of services delivered, and successes and challenges faced in service delivery. Of 59 grantees, 52 were community grants and 7 were state grants. Grantees implemented strategies to identify and support PLAWD by partnering with local service providers and leveraging existing relationships (e.g., meal programs, adult day centers, senior residential communities, friendly visitor programs, transportation services). Grantees offered opportunities for companionship and support for socialization through buddy programs, phone calls, social and wellness programming, and regular home visits. Grantees utilized volunteers, interns, and staff from various disciplines to provide care management or to deliver services. Top challenges reported were difficulty making contact with PLAWD, high levels of stigma associated with dementia, fear of losing independence, and low awareness and a lack of understanding about programs. Findings demonstrate that a variety of services have been successfully provided to PLAWD. However, common challenges are experienced in service delivery. PLAWD need an expansion of services and support, including care management, care monitoring, and socialization. Given the importance of service access for PLAWD, raising awareness of programs, reducing stigma, identifying and reaching PLAWD is vital. Different approaches used and challenges faced between state and community grants are discussed.

